# Metabolomics analysis of dietary restriction results in a longer lifespan due to alters of amino acid levels in larval hemolymph of *Bombyx mori*

**DOI:** 10.1038/s41598-023-34132-9

**Published:** 2023-04-26

**Authors:** Meixian Wang, Yichen Shen, Zhicheng Tan, Ayinuer Yasen, Bingyan Fan, Xingjia Shen

**Affiliations:** 1 Jiangsu Key Laboratory of Sericultural Biology and Biotechnology, College of Biotechnology, Jiangsu University of Science and Technology, Zhenjiang, 212100 Jiangsu China; 2 Key Laboratory of Silkworm and Mulberry Genetic Improvement, Ministry of Agriculture and Rural Affairs, Sericultural Research Institute, Chinese Academy of Agricultural Sciences, Zhenjiang, 212100 Jiangsu China; 3 Department of Plastic Surgery, The First Affiliated Hospital, School of Medicine, Zhejiang University, Hangzhou, 310009 Zhejiang China

**Keywords:** Developmental biology, Physiology, Zoology, Biomarkers

## Abstract

Dietary restriction (DR) has been a very important discovery in modern aging biology research. Its remarkable anti-aging effect has been proved in a variety of organisms, including members of Lepidoptera, but mechanisms by which DR increases longevity are not fully understood. By using the silkworm (*Bombyx mori*), a model of lepidopteran insect, we established a DR model, isolated hemolymph from fifth instar larvae and employed LC–MS/MS metabolomics to analyze the effect of DR on the endogenous metabolites of silkworm, and tried to clarify the mechanism of DR to prolong lifespan. We identified the potential biomarkers by analyzing the metabolites of the DR and control groups. Then, we constructed relevant metabolic pathways and networks with MetaboAnalyst. DR significantly prolonged the lifespan of silkworm. The differential metabolites between the DR and control groups were mainly organic acids (including amino acid), and amines. These metabolites are involved in metabolic pathways such as amino acid metabolism. Further analysis showed that, the levels of 17 amino acids were significantly changed in the DR group, indicating that the prolonged lifespan was mainly due to changes in amino acid metabolism. Furthermore, we identified 41 and 28 unique differential metabolites in males and females, respectively, demonstrating sex differences in biological responses to DR. The DR group showed higher antioxidant capacity and lower lipid peroxidation and inflammatory precursors, with differences between the sexes. These results provide evidence for various DR anti-aging mechanisms at the metabolic level and novel reference for the future development of DR-simulating drugs or foods.

## Introduction

With the increasing problem of global population aging, anti-aging research has become a hot research field. DR with adequate nutrition is the gold standard for delaying aging and extending health span and lifespan in diverse species, including rodents and non-human primates^1–4^. At the same time, it can effectively prevent the deterioration of biological functions and prevent and/or delay the occurrence of aging-related diseases^5–7^.

DR increases lifespan in a broad variety of organisms and improves health in humans. Although increasing evidence suggests that DR works through the key nutrient and stress-responsive metabolic signaling pathways including IIS/FOXO, mTOR, AMPK, sirtuins, NRF2, and autophagy^8–12^, the mechanism by which DR promotes longevity is not fully understood, and the interaction between dietary intake, the microbiome, and the immune system remains poorly described. In addition, the long-term transgenerational consequences of dietary interventions are poorly understood. DR commonly reduces reproduction and long-term DR can be difficult to sustain in humans^4,5,13^. Therefore, it is important to develop new experimental animals with a small size and short life cycle that are more suitable for lifespan research than mice, that are used more frequently in the usual lifespan studies. Invertebrates with simple structures and relatively complete organ differentiation are very good candidates. Currently, only a few invertebrates have been used extensively for lifespan research, namely *Drosophila* and nematodes (e.g. *C.elegans*)^14–16^. Therefore, new experimental animals suitable for aging lifespan research, especially invertebrate models, need to be established.

The silkworm (*Bombyx mori*) is an economically important organism. It has been domesticated for thousands of years and has unique advantages as an experimental invertebrate progeny because it produces a large number of progeny, and has a short life cycle, and it has a clear genetic background that has been used as a model in scientific research for over one century, and a simple living environment. Many studies on the silkworm have also proved that it can be used for aging research^8,17–20^.

Metabolomics is a relatively new high-throughput sequencing technology that has been widely used in various research fields, especially to evaluate the impact of the environment on species^21^. Metabolomics provides information on metabolites in biological cells, tissues, organs, and organisms. These metabolites reflect the life activities of small molecular compounds downstream of the genome at the level of cellular metabolism. They can be measured with various metabolomics methods. The application of metabolomics to silkworm research has increased in recent years^22–25^.

In the previous research, we established a DR model by using the *B. mori* (*Bombyx mori*) Qiufeng strain. One group was submitted to ad libitum feeding (AL) while the other group was submitted to DR. We recorded life indexes such as daily food intake, body weight, age progression, and reproductive capacity to ensure successful establishment of the DR model. Our finding indicates that food restriction can significantly prolong lifespan^20,26^. Moreover, the larvae have sex-limited marks: female larvae have common spots and male larvae have plain spots. Hence, we could separate the larvae sex to evaluate sex-specific changes. The lifespan of female and male in DR model was 144 h and 136 h longer than that in AL group respectively, and the life-prolonging effect was even more obvious after maturity(including pupal and moth stage)^26^. Based on these results, we made the same model in this study and collected hemolymph from fifth instar larvae and performed non-targeted metabolomics using liquid chromatography–tandem mass spectroscopy (LC–MS/MS). Through systematic analysis of differential metabolites, we found that silkworms in the DR group had better antioxidant capacity and lower lipid peroxidation and inflammatory precursors. The results provide experimental evidence and reference for elucidating the biochemical mechanisms by which DR prolongs lifespan, and also lay a theoretical basis to explore lifespan-related biomarkers and to analyze the long-term effects of DR on organisms.

## Materials and methods

### Establishment of the DR silkworm model

The *B. mori* Qiufeng strain was bred and preserved by the Sericulture Research Institute of the Chinese Academy of Agricultural Sciences. It produces diapause eggs when parental eggs are incubated in 25 °C Diapause of eggs were terminated by dipping eggs in a 20% (V/V) HCl solution in 25 °C (for 60 min and thoroughly washed in clean water. The eggs were then incubated in indoor natural light at 25 °C. After hatching, silkworm larvae were reared with fresh mulberry leaves at 25 ± 1 °C. At the second instar stage, the larvae were randomly divided into two groups. The AL group was given food twice a day and allowed uninterrupted feeding during the entire day. The DR group was given mulberry leaves once a day and allowed to feed for 16 h. There was no food available for the other 8 h. The DR group feeding time was 66.6% of that of the AL group, which meets the DR standard of other animal models (60%)^27,28^. From the fourth instar, females and males of each groups were reared separately according to their body marks, with three replicates for each group and 30 larvae for each repetition.

### Sample preparation for LC–MS/MS

Fifth instar male and female larvae from the AL and DR were wiped with 75% alcohol and hemolymph was collected from the caudal legs. Eighty larvae were used for each experimental group. And 300 μL hemolymph was collected from each 10 larvae as one sample, totally 8 hemolymph samples of each experimental group were used for LC–MS/MS. The hemolymph was collected in 1.5 mL centrifuge tubes that contained phenylthiourea, then immediately transferred to -80° for freezing. Samples were prepared as follows:

**Table:** 

Groups	AL♀	AL♂	DR♀	DR♂
Quantity * volume	8*300 μL	8*300 μL	8*300 μL	8*300 μL

All samples were thawed at 4 °C. Then, 200 μL of each sample was combined with 800 μL of methanol; the tube was shook for 60 s, and then mixed well. The samples were centrifuged at 12,000 rpm for 10 min at 4 °C. The supernatant was transferred to a new 1.5 mL centrifuge tube, then concentrated to dryness in vacuo. Dried samples were reconstituted with 300 µL of 80% methanol and filtered with a 0.22 μm membrane. These samples were subjected to LC–MS.

Twenty microliters of each sample was mixed to form a quality control (QC) sample, which was used to correct deviations of the analysis results and errors caused by the analytical instrument itself. The remaining samples were used for LC–MS on-machine detection.

### LC and MS conditions

For LC, a Thermo Ultimate 3000 instrument and ACQUITY UPLC^®^ HSS T3 1.8 µm (2.1 × 150 mm) column were used. The autosampler temperature was 4 °C, the flow rate was 0.25 mL/min, and the column temperature was 40 °C. Two microliters of the sample was injected for gradient elution. The mobile phase for the positive ion mode was 0.1% formic acid water (A) and 0.1% formic acid acetonitrile (B). The mobile phase for negative ion mode was 5 mM formic acid amine water (C) and acetonitrile (D). The gradient elution program was 0–1 min, 2% B/D; 1–9 min, 2–50% B/D; 9–14 min, 50–98% B/D; 14–15 min, 98% % B/D; 15–15.5 min, 98–2% B/D; 15.5–17 min, 2% B/D.

For MS, a Thermo Q Exactive Focus instrument with an electrospray ion source (ESI) was used in positive and negative ion ionization modes. The positive ion spray voltage was 3.80 kV, the negative ion spray voltage was 2.50 kV, the sheath gas 45 bar, and auxiliary gas was 15 bar. The capillary temperature was 325 °C, the full scan was performed with a resolution of 70 000, the scan range was 81–1000, the secondary fragmentation was performed by high-energy collisions (HCD), the collision voltage was 30 eV, and the unnecessary MS/MS information was removed by dynamic exclusion.

### Data analysis

The acquired LC–MS/MS data raw files were imported into Proteowizard software (v3.0.8789). Then, the acquired raw data were converted into the mzXML format (xcms input file format). The XCMS package of R (v3.3.2) was used to identify, filter, and align peaks. The main parameters were bw = 5, ppm = 15, peakwidth = c (10, 20), mzwid = 0.015, mzdiff = 0.01, and method = centWave. The data matrix was obtained including the mass-to-charge ratio (m/z), retention time (rt), peak area (intensity), and other information. To enable data of different magnitudes to be compared, peak areas were batched normalized and then imported into SIMCA-P 14.1 (Umetrics, Sweden) for principal component analysis (PCA), partial least squares discriminant analysis (PLS-DA), and orthogonal partial least squares discriminant analysis (OPLS-DA). The differential variables that contributed the most were determined by variable importance in projection (VIP) > 1 in the S-plot and *p* < 0.05 on an independent samples t-test. Finally, the differential variables were identified through the Human Metabolome Database (HMDB) database (https://hmdb.ca/) combined with secondary fragment ions. The differential metabolite data obtained from the identification was used to carry out pathway enrichment analysis with MetaboAnalyst5.0 (https://www.metaboanalyst.ca/).

## Results

### Effects of DR on the life index of silkworms

**Figure 1:**
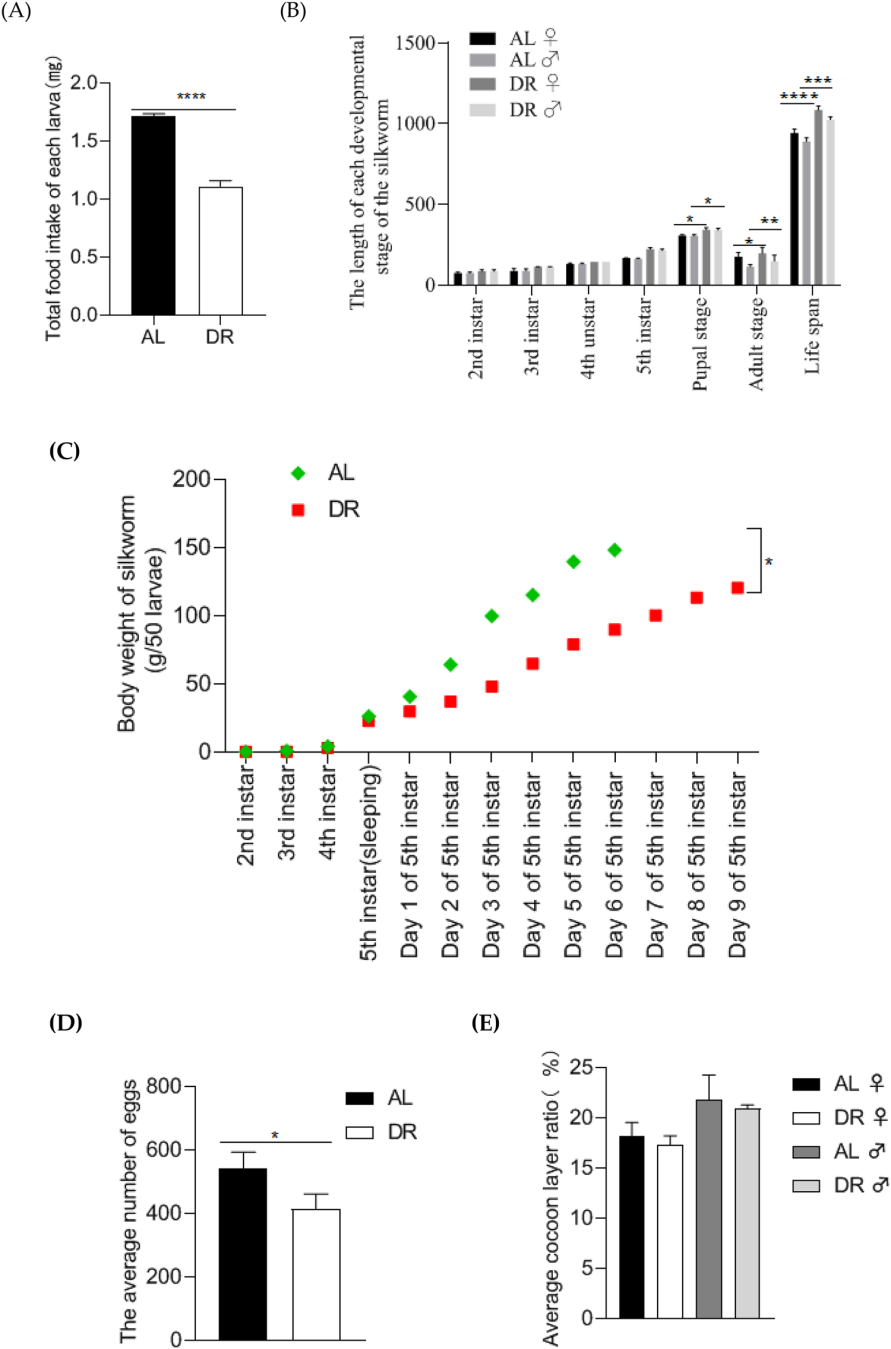
Effects of DR on the Life Index of Silkworms. (**A**) The feeding amount in DR and AL group; (**B**) Comparison of the duration of each instar of *Bombyx mori* in DR and AL group; (**C**) Body weight of silkworm larvae at different stages,the chart shows the weight per 50 larvea; (**D**) Comparison of the number of eggs laid by females in DR and AL group; (**E**) Comparison of cocoon layer rate between DR and AL group.

By controlling the feeding time, the feeding amount of DR group was 65.07% of that in AL group (Fig. 1A).The entire lifespan (including larval, pupal and adult stage) lasted significantly longer (*p* < 0.05), specifically 143 and 137 h longer for females and males, respectively, compared with the AL group, and the extension of survival time was more significant in the pupal and adult stage (Fig. 1B). Although diet restriction caused the decrease of body weight in the later period of silkworm life (Fig. 1C), and the number of eggs laid by females declined (Fig. 1D), it had no obvious negative effect on cocoon layer rate (Fig. 1E). These results indicate that rational diet restriction can significantly prolong life expectancy with little impact on economic and life indexes.

### Metabolites differ significantly between DR and AL groups

**Figure 2:**
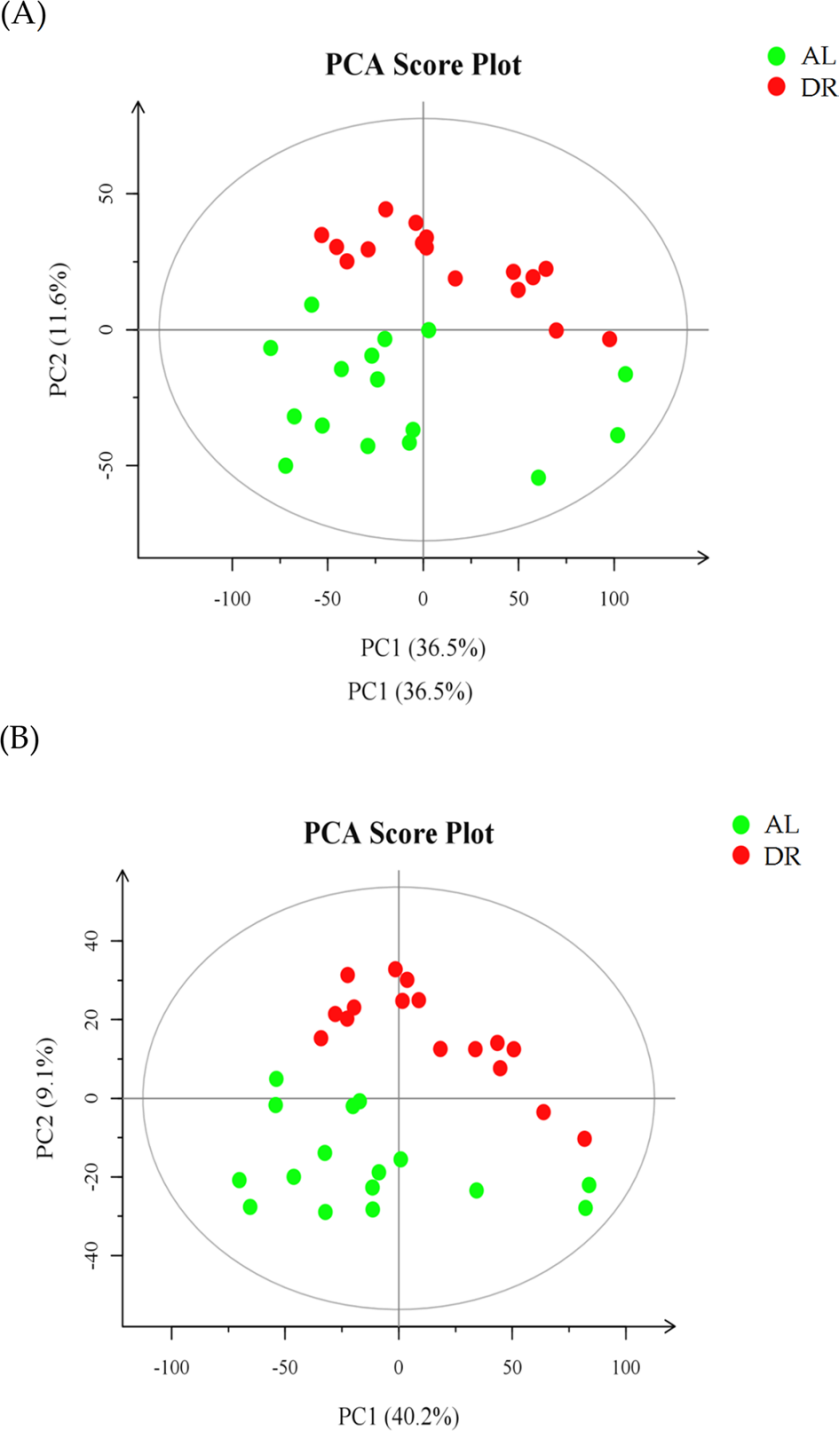
Scatter plot of the principal component analysis. (**A**) positive ion mode; (**B**) negative ion mode. *Note* AL, ad libitum feeding; DR, Dietary restriction.

PCA generated new characteristic variables by linear combination of metabolite variables according to a certain weight, and categorized each group of data through the main new variables (principal components) to remove poor repeatability (outlier samples) and abnormal samples. This approach intuitively reflects the distribution of each sample in the mathematical model space. From the PCA score plot, the samples were obviously aggregated into groups, indicating a similar composition and concentration of the variables/molecules in the same group of samples (Fig. 2).

PLS-DA can measure the impact strength and explanatory power of expression patterns of metabolites to classify and discriminate samples, thereby assisting to screen metabolic markers. The PLS-DA model revealed that the DR and AL groups could be distinguished in the positive and negative ion modes (Supplementary Fig. 1).

We used OPLS-DA regression to establish the relationship between metabolite expression and sample category, to predict the sample group. There was good discrimination between the DR and AL groups in the positive and negative ion modes (Supplementary Fig. 2).

### Potential biomarkers for DR-induced lifespan extension in silkworms

**Figure 3:**
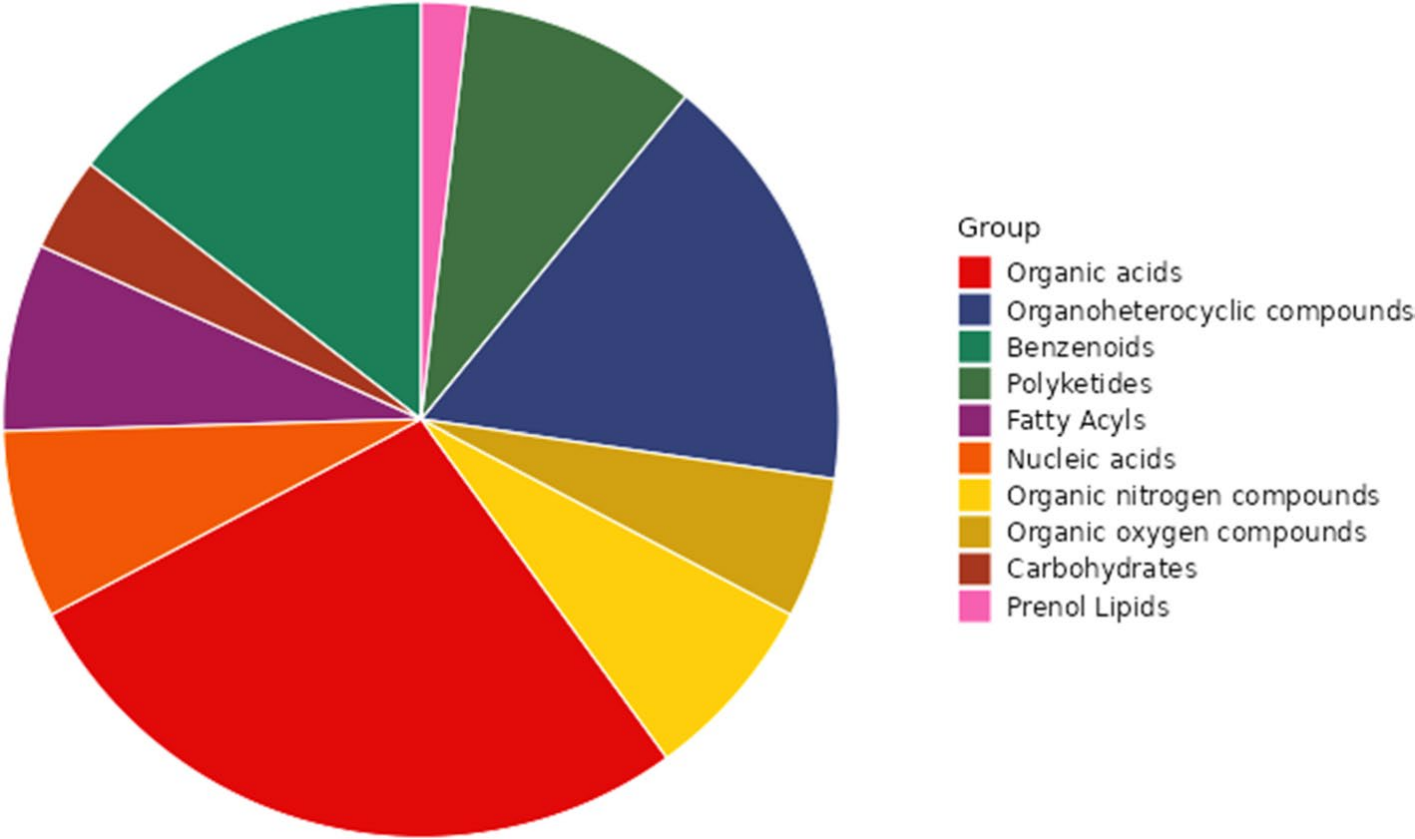
Classification of differential metabolites.

Biomarkers can provide important information regarding the state of metabolic pathways. Identifying biomarkers would allow one to analyze comprehensively the mechanism by which DR increases lifespan. The effect of DR on the hemolymph metabolites of silkworm was obvious.Among the detected metabolites, 1042 metabolites were upregulated and 1,692 were downregulated compared with the AL group (Supplementary Fig. 3).We identified 80 differential metabolites between the DR and AL groups (based on *p* < 0.05 and VIP ≥ 1 for the first component of OPLS-DA). Most of these differential metabolites are organic acids, of which amino acids, peptides, and their derivatives represent the largest proportion (53.3%, Fig. 3, Supplementary Table 1). The metabolites with the most significant differences are amino acids (including peptides and their derivatives, such as citrulline, L-glutamine, 5-oxo-L-proline, beta-alanine, N-acetyl-L-phenylalanine, L-phenylalanine, and L-histidine),the levels of 17 amino acids were significantly changed in the DR group(Table 1), followed by other organic acids (2-oxoglutaric acid, 3-hydroxy-3-methylglutaric acid, 5-aminosalicylic acid, and aminohippuric acid), amines (orotidine, ribothymidine, guanosine, 2-methylguanosine, uridine, and inosine), and biogenic amines (N-omega-acetylhistamine, histamine, putrescine, thymine, epsilon-caprolactam, and cyclohexylammonium) (Supplementary Table 1, Supplementary Fig. 4). Organic acids, amines, and biogenic amines are metabolized vigorously in silkworm hemolymph, indicating that the changes in these metabolites are inseparable from DR.

**Table 1:** J 17 amino acids that level changed significantly in the DR group.

Amino acid	Mean_AL	Mean_DR	Fold change	Log2 (FC)	*p* value	FDR
L-Phenylalanine	19,200,919.35	14,296,314.96	0.74456	− 0.42553	3.01E−06	0.00030349
Citrulline	98,769,293.16	206,759,036.3	2.0934	1.0658	3.32E−06	0.000321484
L-Glutamine	4,944,020,962	5,896,912,379	1.1927	0.25427	0.000189198	0.004417884
5-Oxo-L-Proline	3,318,487,561	3,963,115,740	1.1943	0.25611	0.000237059	0.004974341
L-Histidine	10,976,341,701	9,151,456,845	0.83374	− 0.26232	0.000343632	0.006361452
beta-Alanine	512,919,211.5	614,938,175.3	1.1989	0.26171	0.000501213	0.007897442
Glycine Betaine	5,676,864,188	9,211,875,930	1.6227	0.6984	0.002378735	0.019590033
3-Hydroxy-DL-kynurenine	210,599,831.8	118,910,653.1	0.56463	− 0.82463	0.002863303	0.022173951
ADMA	2,591,734,286	1,985,470,489	0.76608	− 0.38444	0.009657901	0.048930094
L-Asparagine	421,067,355.7	530,654,290.9	1.2603	0.33372	0.011296623	0.054180258
L-Kynurenine	69,673,758.07	46,561,638.19	0.66828	− 0.58147	0.02034124	0.078170374
L-Isoleucine	11,010,270,065	7,834,365,254	0.71155	− 0.49096	0.022427126	0.083545406
N-Acetyl-L-Phenylalanine	676,923.76	1,475,764.267	2.1801	1.1244	0.000125896	0.003624221
PS(18:1(9Z)/0:0)	5,570,516.541	3,811,703.205	0.68426	− 0.54738	0.004071645	0.028608243
N-Acetylproline	6,140,567.251	4,537,858.364	0.739	− 0.43636	0.005675749	0.035375506
L-Leucine	7,961,590.504	5,914,799.329	0.74292	− 0.42873	0.015074813	0.064331681
4-Hydroxy-L-Proline	7,724,893.857	5,715,649.62	0.7399	− 0.4346	0.022334042	0.084458617

*FDR* false discovery rate.

### Metabolic pathway analysis of potential biomarkers

**Figure 4:**
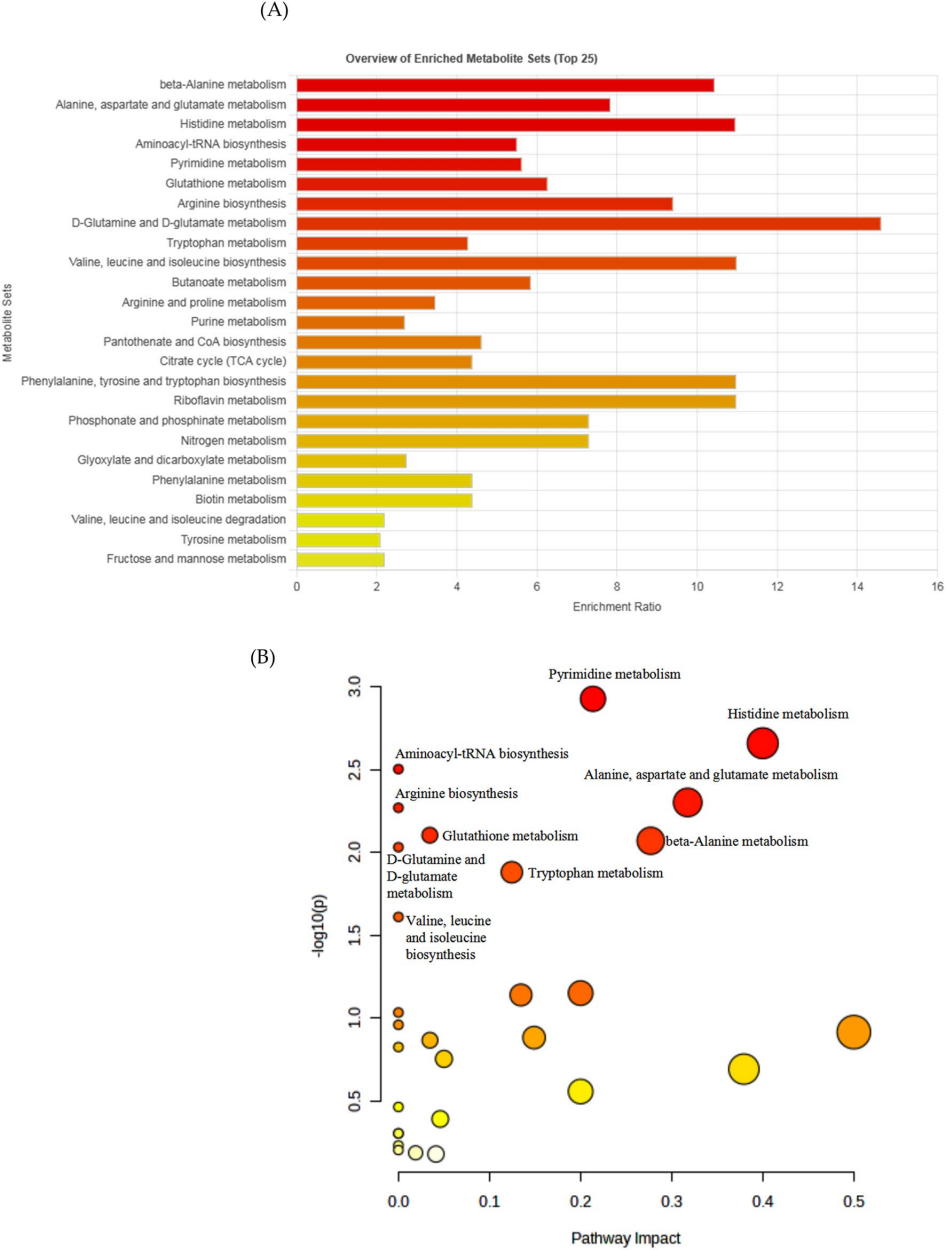
Enrichment of differential metabolites. (**A**) Metabolism pathway enrichment of differential metabolites; (**B**) Pathway impact of differential metabolites.

We used the MetaboAnalyst database to analyze the differential metabolites between the DR and AL groups. We integrated the raw p values, the Holm p value, false discovery rate (FDR), and impact value, and we evaluated the importance of related metabolic pathways. We obtained comprehensive data of metabolic pathways by removing metabolites with a raw and Holm *p* > 0.1 and false positive rate > 10% (0.1). In the DR group, the differential metabolites were mainly concentrated in amino acid metabolism, carbohydrate metabolism, and energy metabolism (Fig. 4A), and the differential metabolites showed significant differences in multiple metabolic pathways (Fig. 4B). In addition, the tricarboxylic acid (TCA), pyrimidine and purine metabolism, biotin metabolism, and riboflavin metabolism showed significant changes. Notably, amino acid metabolism accounted for more than 50% of the total metabolic pathways, followed by carbohydrate metabolism. Combined with the results of cluster analysis, DR altered the ratio of amino acids and increased amino acid utilization, which may be the most important reason for the increased longevity of silkworms submitted to DR. We confirmed that DR could promote fatty acid oxidative metabolism by intervening in mitochondria, thereby prolonging lifespan. The TCA cycle was activated after DR, and this change might be the result of mitochondrial activity.

**Figure 5:**
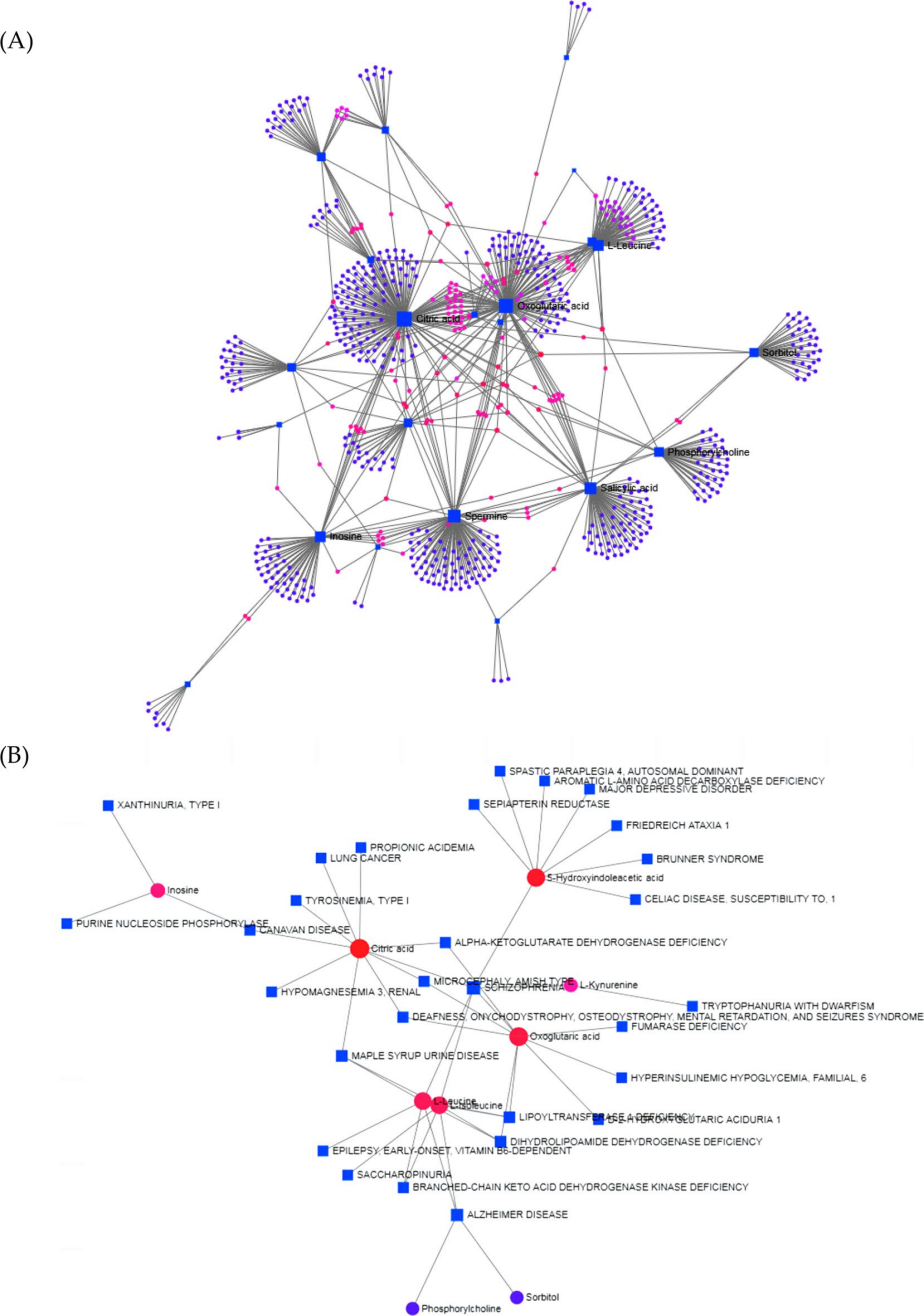
Analysis of interaction between different metabolites and diseases, genes. (**A**) Gene–metabolite interaction networks. (**B**) Metabolite–disease interaction.

We also analyzed the potential relationship between differential metabolites, genes (Fig. 5A, Attachment 1), and diseases (Fig. 5B, Attachment 1). We found that the differential metabolites are involved in a variety of geriatric diseases and multiple lifespan-related genes. Hence, DR may modulate lifespan genes and promote anti-aging processes.

### DR Effects on metabolism in each sex

**Figure 6:**
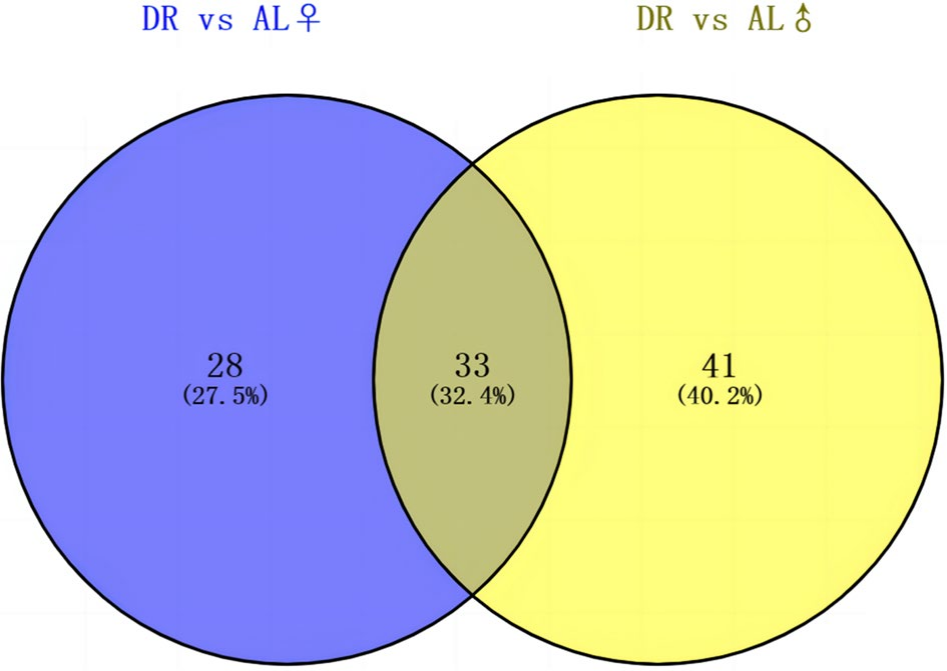
Venn diagram of differential metabolites in the male and female groups. AL, ad libitum feeding; DR, dietary restriction.

We found that metabolite differences in silkworm hemolymph were not only affected by DR, but were also closely related to silkworm sex. The lifespan extension induced by DR was more obvious in female silkworms than that in male indicating that sex is inseparable from the effects of DR. To analyze the metabolic differences between female and male silkworms, we performed a multivariate analysis based on metabolomics data, and we separated the two groups of differential metabolites based on sex. We found 41 unique differential metabolites in the male group and 28 unique differential metabolites in female silkworms (Fig. 6, Supplementary Tables 2 and 3), further indicating that sex has a great effect on metabolites in the hemolymph. Thirty-three differential metabolites appeared simultaneously in the two experimental groups, indicating that these metabolites are closely related to aging. Of these metabolites, 25 showed similar trends (Supplementary Fig. 5A, Supplementary Table 4) and the other 8 showed the opposite trend (Supplementary Fig. 5B, Supplementary Table 4). The differential metabolites with the identical trend are predominantly amino acids (Supplementary Fig. 6A). The unique differential metabolites are predominantly amino acids and glycerophospholipids in females (Supplementary Fig. 6B) and predominantly pyrimidine, amino acids, pantothenic acid, and coenzyme A in males (Supplementary Fig. 6C). These findings make sense: females lay eggs, so they have higher amino acid and energy needs than males.

## Discussion

As the global population continues to age, there has been increased attention of health problems caused by aging and aging-related diseases. Over the past decade, research on aging and its accompanying diseases has accelerated markedly. DR is a powerful way to prolong lifespan and is one of the most recognized and successful anti-aging methods. However, how it induces metabolic changes associated with aging has been poorly studied, and the focus has mainly been on vertebrates^29–32^. Studies have shown that organisms respond to DR stimuli in complex and unpredictable ways. At this stage, the available research is still insufficient to determine how DR prolongs lifespan and allows organisms to resist aging-related problems. Several pathways known to be involved in anti-aging mechanisms (e.g., free radical generation, formation of advanced glucose oxidation end products, lipid peroxidation, and inflammatory responses) are closely related to metabolism. Therefore, metabolite analysis can be used to discover potential aging biomarkers and to test various hypotheses of the mechanisms by which DR prolongs lifespan by determining the metabolic changes induced by DR. By measuring and mathematically modeling changes in the levels of hemolymph metabolites in the invertebrate silkworm, we have provided new insights into the mechanisms by which DR prolongs life. At the same time, our non-targeted metabolic analysis covers the whole set of metabolic pathways, and our results provide detailed metabolic characteristics of biological systems responding to DR.

### DR induced marked changes in amino acids

Amino acids are essential nutrients for silkworms to maintain body functions and growth. They compose most enzymes and some hormones and other active substances that regulate the physiological activities of silkworms^33^. In the DR group, the total amount of amino acids in the silkworm diet decreased, and there were specific reductions in isoleucine and arginine but increases in alanine, asparagine, and other amino acids. Some specific amino acids have established effects on lifespan—for example, methionine restriction increases lifespan in *Drosophila* by downregulating TOR signaling in the state of low amino acid concentrations^34^. Consistently, tryptophan restriction increases lifespan in mice and Evans rats, and dietary methionine restriction in rats and mice reduces mitochondrial oxygen free radicals, blood glucose, insulin, and IGF-1 levels, increasing average and maximum lifespan^35,36^. These studies indicate a key role for proteins in the regulation of lifespan in *Drosophila* and mammals.

In this study, many amino acid metabolic pathways changed significantly after dietary restriction (Fig. 4). For example, the metabolic changes of the three branched-chain amino acids—valine, leucine, and isoleucine—are closely related to longevity and aging^37^ suggesting that lifespan extension in DR may also be the result of changes in branched-chain amino acids. In addition, compared with the AL group, the content of citrulline increased by 2.09 times. As an important part of the liver, citrulline can help the immune system resist infection and increase energy, indicating that DR may prolong lifespan by improving immunity. The types and quantities of various amino acids in the silkworm follow a certain strict ratio. Too little or too much intake of amino acids will hinder protein synthesis, thus affecting their lifespan^33^. The essential amino acids required by the silkworm are the same as those required by other insects or higher animals^38^, among which tryptophan and methionine are the main limiting amino acids^33^. We found no differences in the contents of these two limiting amino acids in the hemolymph, suggesting that although the AL group ingested more amino acids, they did not utilize amino acids better than the DR group. Therefore, changes in the ratio of amino acids and utilization by DR may also be an important reason for prolonging life^39^. Metabolomics has advanced our understanding of the complex relationship between amino acid metabolism and lifespan to identify the role of specific amino acids in maintaining health and the impact on lifespan. This technique also facilitates the study of amino acid functions, metabolic regulation, and safety, and serves as a reference for the development and design of dietary regimens restricting specific amino acids.

### DR altered carbohydrate metabolites

The TCA cycle is a crucial metabolic pathway in silkworms. It contains and affects a variety of metabolites in the body, and has a wide and profound connection with aging^40^. TCA metabolites such as citric acid were increased in the DR group, indicating an increase in TCA cycle activity in this group. Studies have shown that a decrease in the levels of circulating TCA metabolites reduces the number of cells, and NAD + concentrations decrease during aging; these changes increase the body’s oxidative stress response. Accompanied by the functional decline of these cellular signals, age-related oxidative stress and inflammation increase gradually, but restoring NAD + levels in the body can extend lifespan to a certain extent^41^. In other words, DR may have facilitated a better energy supply and antioxidant capacity by activating the TCA cycle, indicating that silkworms submitted to DR have a better health state and thus prolonged lifespan. In addition, the TCA cycle intermediate citrate may play a role in amino acid and fatty acid metabolism^42^. Interestingly, elevated citrate, which inhibits phosphofructokinase, and glycolysis inhibitors such as 2-deoxyglucose have been proposed as viable DR mimics^43^.

Organisms respond when energy supply levels are low and try to delay aging and enhance resistance to aging pathologies. Determining the mechanisms behind this response could provide a way to treat a variety of diseases and prolong life in one fell swoop. Mitochondria are cellular energy factories. The enzymes and proteins distributed on the inner and outer membranes of mitochondria are important components of the oxidative respiratory chain and participate directly in the energy production process^44^. Metabolomics revealed that the silkworm TCA cycle was activated after DR, and this change was apparently the result of mitochondrial activity.

### DR altered biogenic amines

We detected changes in biogenic amines such as spermine, putrescine, spermidine, histamine, and tyramine. Except for histamine, the rest showed a downward trend. Appropriate amounts of biogenic amines can promote the normal physiological activities of organisms, while excessive intake can cause adverse reactions^45^. Biogenic amines are mainly formed by microbial strains producing amino acid decarboxylase catalyzing the decarboxylation of free amino acids. Therefore, microbial strains producing amino acid decarboxylase are a key factor that limits the content of biogenic amines^46^. The intestinal flora metabolize the ingested amino acids into biogenic amines, which enter the hemolymph through the intestinal wall^33^. Therefore, the reduced biogenic amine content suggests that DR can reduce the accumulation of biogenic amines by inhibiting microbial growth and metabolic activity.

The gut microbiota has a marked impact on lifespan. Studies have shown that the gut microbiota induces symbiosis in the host after a problem with the immune system, thereby promoting the occurrence of aging-related diseases^29^. In short, the gut microbiota indirectly promotes aging-related diseases. Diet has a huge impact on the intestinal microbes of silkworm, which indicates that DR induces changes in the intestinal flora and changes the intestinal microecology of silkworms, thereby reducing the occurrence of inflammation, stabilizing the immune system, and prolonging silkworm lifespan.

### DR altered other metabolites

DR also enriched nucleic acid and vitamin metabolism in silkworms. Elevated purine/pyrimidine metabolites (1.63-fold increase for thymine and 1.39-fold increase for hypoxanthine) support RNA synthesis and recombination, which also confirms that DR may prolong lifespan by epigenetic mechanisms^47^. Vitamins increase the lifespan of various organisms primarily by acting as antioxidants^42^. However, vitamins such as biotin, riboflavin, ascorbic acid, and choline all showed a downward trend. While vitamins are generally considered to have health benefits, there is growing evidence that they also shorten lifespan. The antioxidant function of ascorbic acid reduces the long lifespan conferred by mildly increased reactive oxygen species levels in *C. elegans*^48^. Moreover, vitamin A supplementation for 6 months increased C-reactive protein, an inflammatory factor^49^. These studies suggest that the conventional wisdom that vitamins promote health benefits and slow aging should be applied with caution. In addition, we must consider the possibility that different species have different physiological responses or dietary signal responses, and the mechanism of action of many vitamins in silkworm remains to be proved by further studies.

### DR promoted sex-specific metabolite changes

The impact of sex on life expectancy is an issue that cannot be ignored^50^. In this study, the lifespan extension caused by DR was more obvious in female silkworms than that in male silkworms, indicating that the effect of DR on lifespan extension was sex-specific, and the effect was better in female. Among the 33 common differential metabolites, 8 showed opposite trends in the sexes, namely L-cystathionine, L-tyrosine, phenethylamine, L-arginine, adipic acid, dulcitol, L-lysine, and L-piperic acid (Supplementary Fig. 5). Most of these metabolites are amino acids and their intermediates. Differences in cystathionine are related to egg formation, and female silkworms require more of it than males. Cystathionine decreased 3.23 times in males and increased 3.24 times in females after DR treatment, indicating that the utilization of specific amino acids was changed in a sex-specific manner.

## Conclusion

We employed LC–MS/MS-based metabolomics to evaluate DR-induced changes in hemolymph metabolites. DR altered organic acids and amines, especially the ratio and utilization of amino acids, indicating that the DR group has a higher amino acid utilization, a healthier gut flora, and a more active energy response than the AL group. DR promotes better antioxidant capacity and lower lipid peroxidation and inflammatory precursors, which may be the reason for the lifespan extension. Restricting the content of certain amino acids and maintaining adequate vitamin levels will be key considerations in the future development of food and medicine for aging. We also explored some lifespan-related biomarkers and genes based on the relationship between the metabolome and genes and diseases. Our future research will explore these biomarkers and genes and evaluate the long-term effects of food restriction on the body.

## Supplementary Information

Supplementary Information.

Supplementary Figures.

Supplementary Tables.

## Data Availability

Most of the analytical data is provided in the article and supplementary data. More original datasets used and/or analysed during the current study available from the corresponding author on reasonable request.
