# Supplementary material for: Metabolomics analysis of dietary restriction results in a longer lifespan due to alters of amino acid levels in larval hemolymph of Bombyx mori
Source: Sci Rep. 2023 Apr 26;13:6828. doi: 10.1038/s41598-023-34132-9 (PMC10133320; doi:10.1038/s41598-023-34132-9)
Supplement: Supplementary file 2 — Supplementary Figures. [file 41598_2023_34132_MOESM2_ESM.docx]

**Metabolomics Analysis of Dietary Restriction Results in a Longer Lifespan Due to Alters of Amino Acid Levels in Larval Hemolymph of *Bombyx mori***

Meixian WANG ^1,2†^, Yichen SHEN ^3†^, Zhicheng TAN ^1†^, Ayinuer YASEN^1,2^, Bingyan FAN^1,2^, Xingjia SHEN ^1,2^*

1. Jiangsu Key Laboratory of Sericultural Biology and Biotechnology, College of Biotechnology, Jiangsu University of Science and Technology, Zhenjiang 212100, Jiangsu, China
2. Key Laboratory of Silkworm and Mulberry Genetic Improvement, Ministry of Agriculture and Rural Affairs, Sericultural Research Institute, Chinese Academy of

Agricultural Sciences, Zhenjiang 212100, Jiangsu, China

3. Department of Plastic Surgery, the First Affiliated Hospital, School of Medicine，Zhejiang University, Hangzhou 310009, Zhejiang, China

^†^These authors contributed equally to this work

*corresponding author: shenxjsri@163.com

**Corresponding author:**

SHEN Xingjia (Ph.D.)

Jiangsu University of Science and Technology

College of Biotechnology

Key Laboratory of Silkworm and Mulberry Genetic Improvement

212100 Zhenjiang

Email:shenxjsri@163.com`

(A)


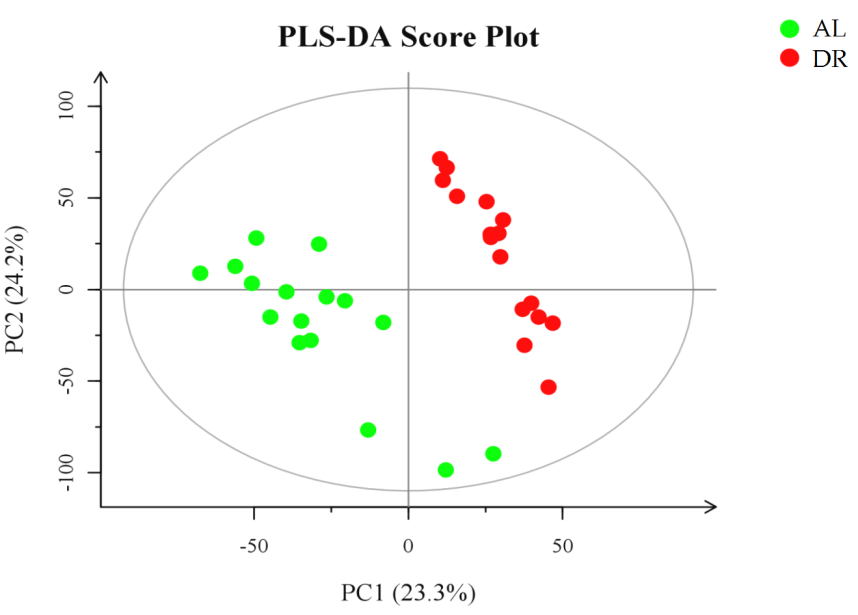


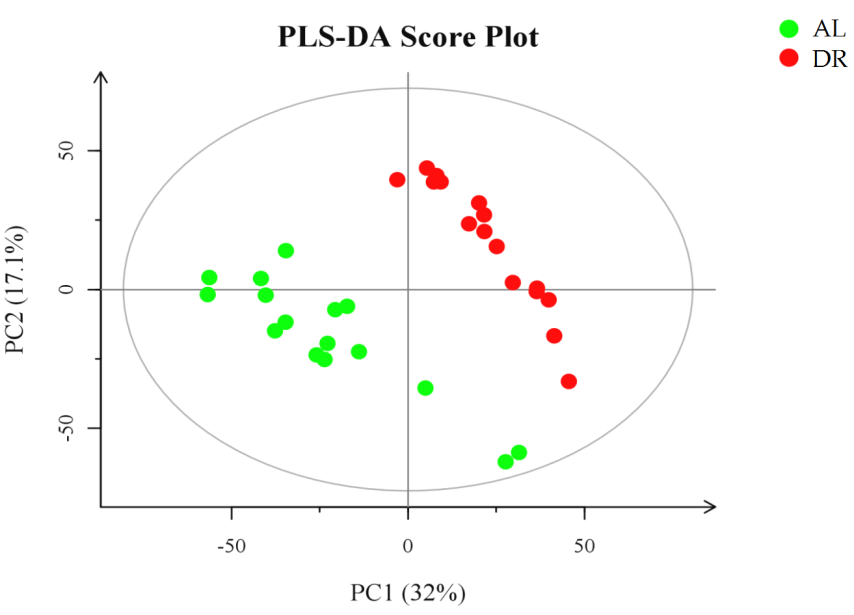
(B)

**Supplementary Figure 1 Scatter plot of the partial least squares discriminant analysis**

(A) positive ion mode; (B) negative ion mode.

Note AL: ad libitum feeding ; DR:Dietary restriction.


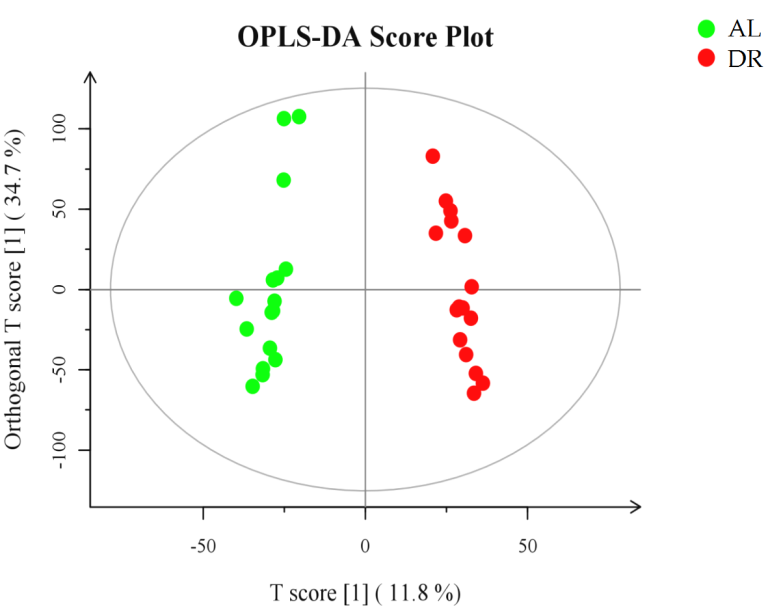
(A)


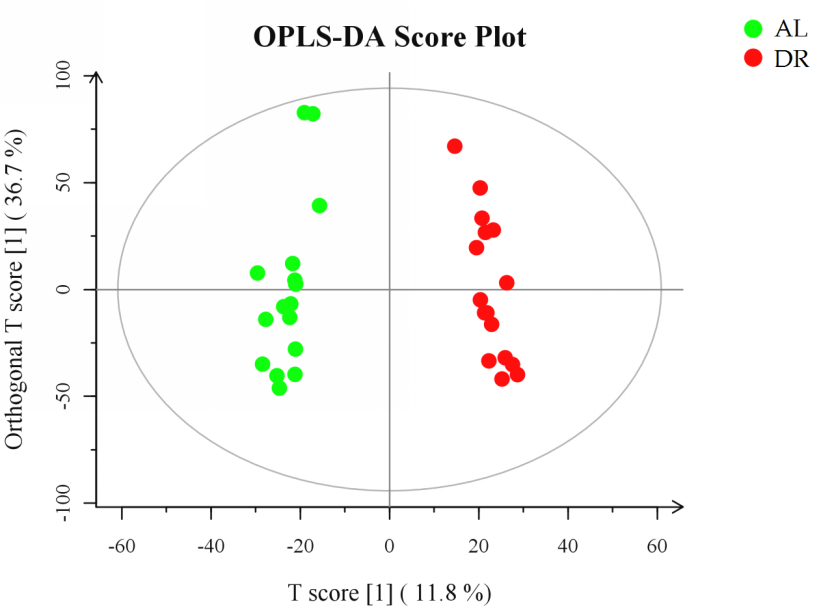
(B)

**Supplementary Figure 2 Scatter plot of the orthogonal partial least squares discriminant analysis**

(A) positive ion mode; (B) negative ion mode.

Note AL: ad libitum feeding; DR:Dietary restriction.


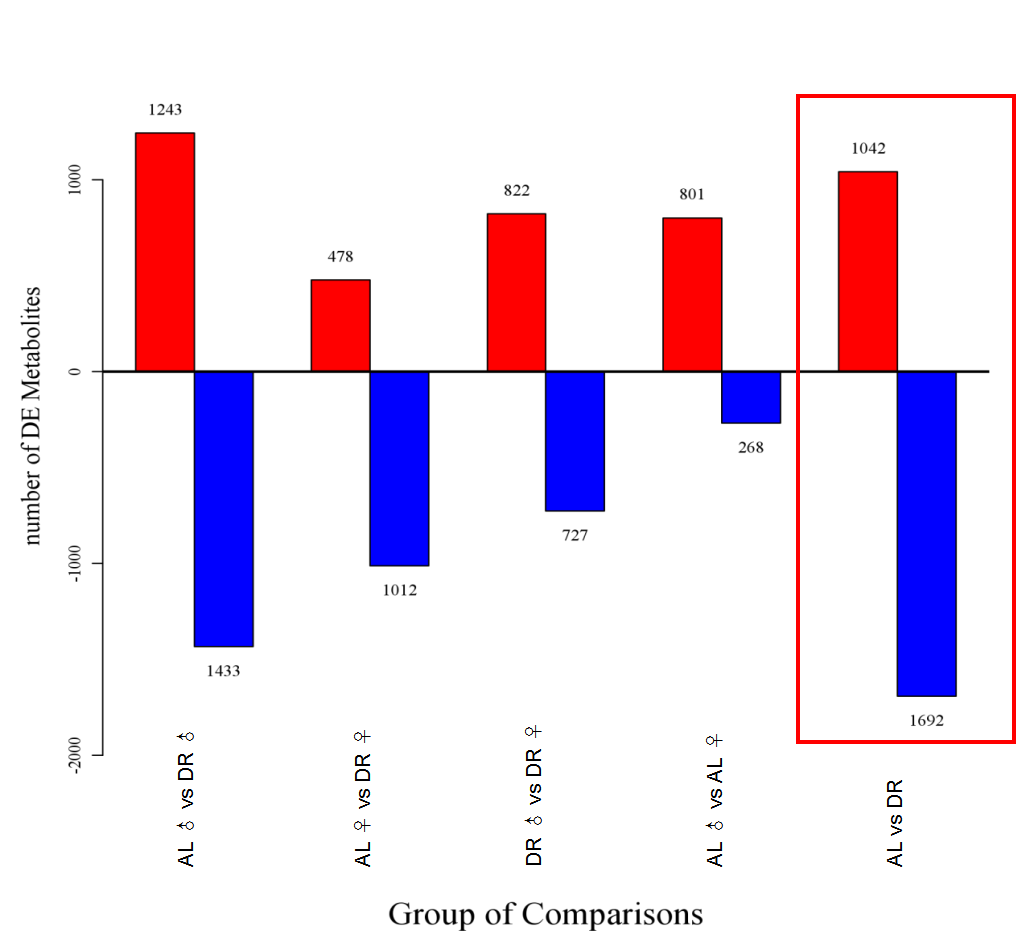


**Supplementary Figure 3 The number of differential metabolites between the dietary restriction (DR) and *ad libitum* feeding (AL) groups**

Note ♂,male；♀,female.


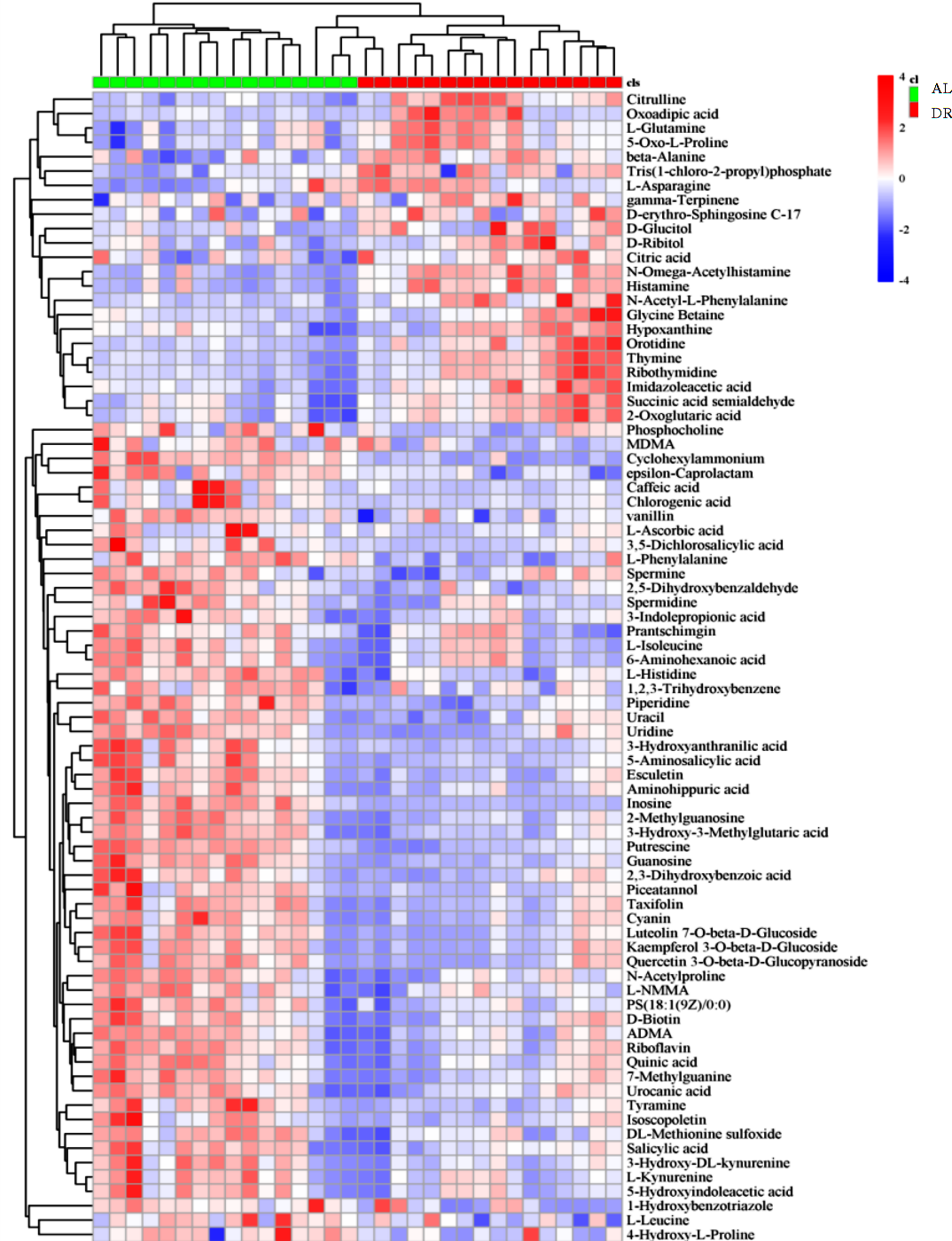


**Supplementary Figure 4 Heatmap of differential metabolites in experimental groups**

Agglomerate hierarchical clustering was used in the experiment: each object is grouped into one type, which is combined to form larger and larger objects until it is terminated. Scaling the data set by the pheatmap package(v 1.0.12) [(https://www.rdocumentation.org/packages/](https://www.rdocumentation.org/packages/pheatmap/versions/1.0.12)

[pheatmap/ versions/1.0.12)](https://www.rdocumentation.org/packages/pheatmap/versions/1.0.12) in R (v3.3.2) [(https://cran-archive.r-project.org/bin/windows/](https://cran-archive.r-project.org/bin/windows/base/old/3.3.2/)

[base/old/3.3.2/)](https://cran-archive.r-project.org/bin/windows/base/old/3.3.2/) results in this hierarchical clustering diagram of relative quantitative values of metabolites: The size of the relative content in the diagram is shown by color differences, with columns representing samples and rows representing metabolites.

Note AL: ad libitum feeding ; DR:Dietary restriction.


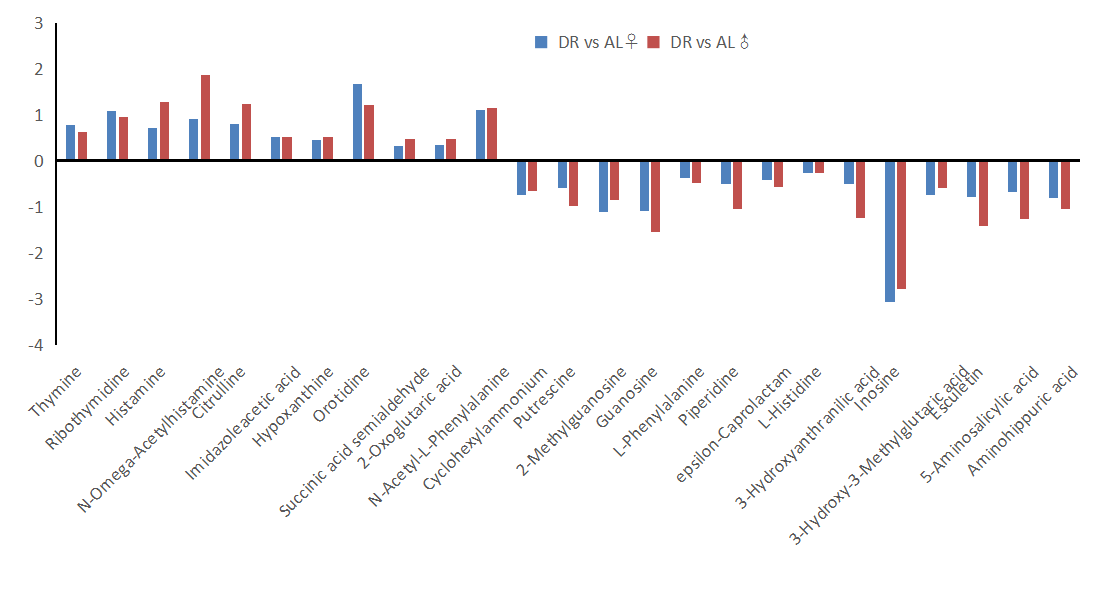
(A)


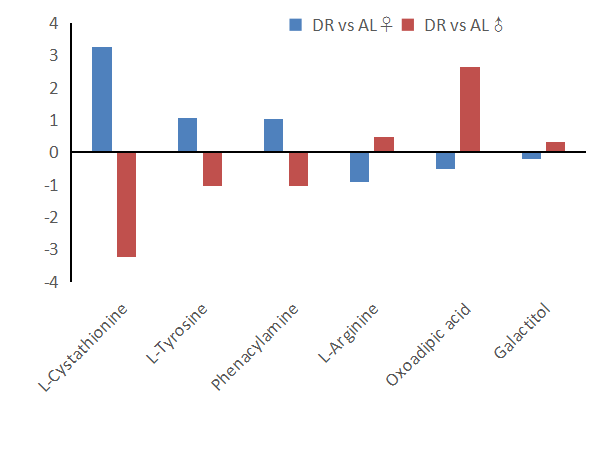
(B)

**Supplementary Figure 5 Comparison of differential metabolites in female and male silkworms**

(A) Metabolites with opposite trends of common differential metabolites; (B) Metabolites with the same trend of common differential metabolites.

AL, ad libitum feeding; DR, dietary restriction.

(A)


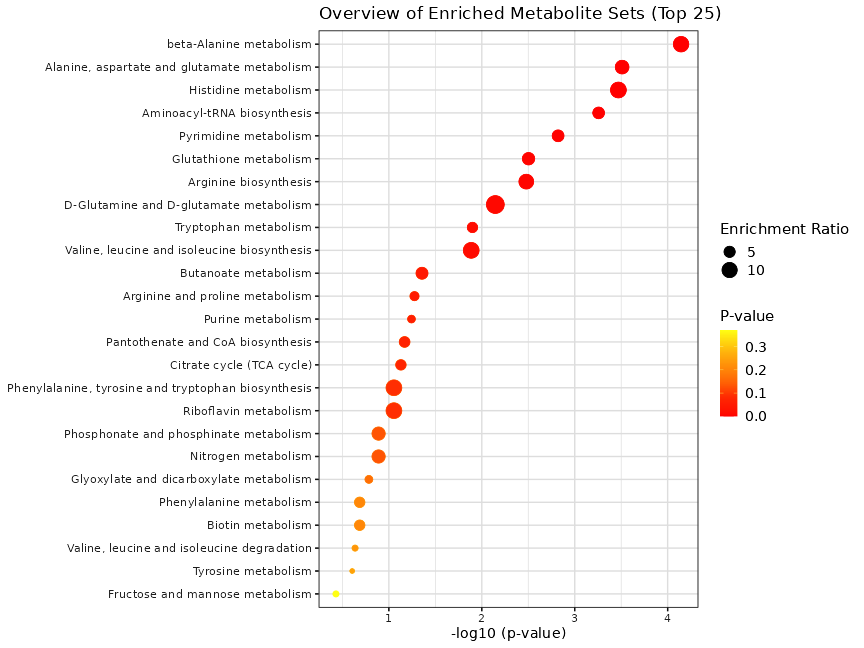


(B)
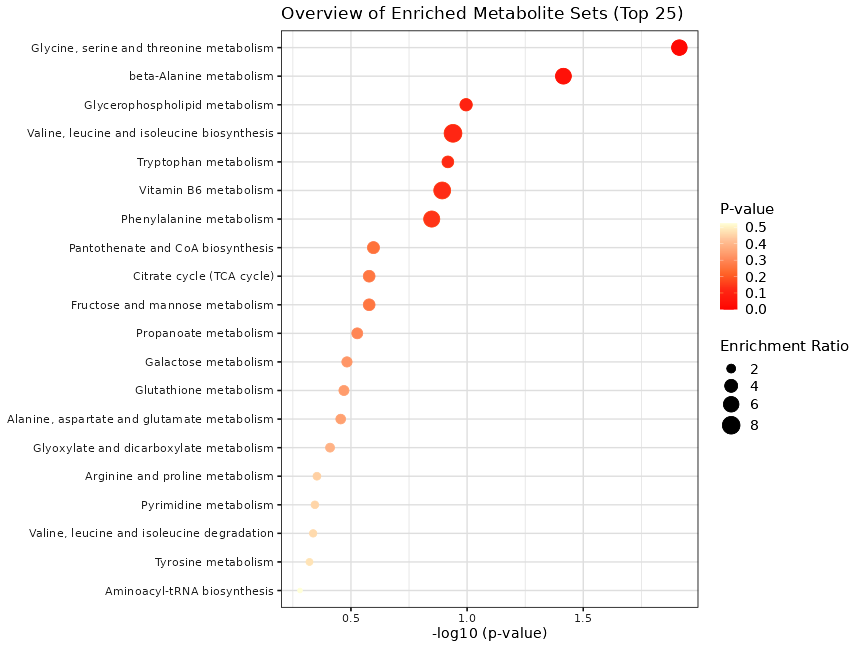


(C)


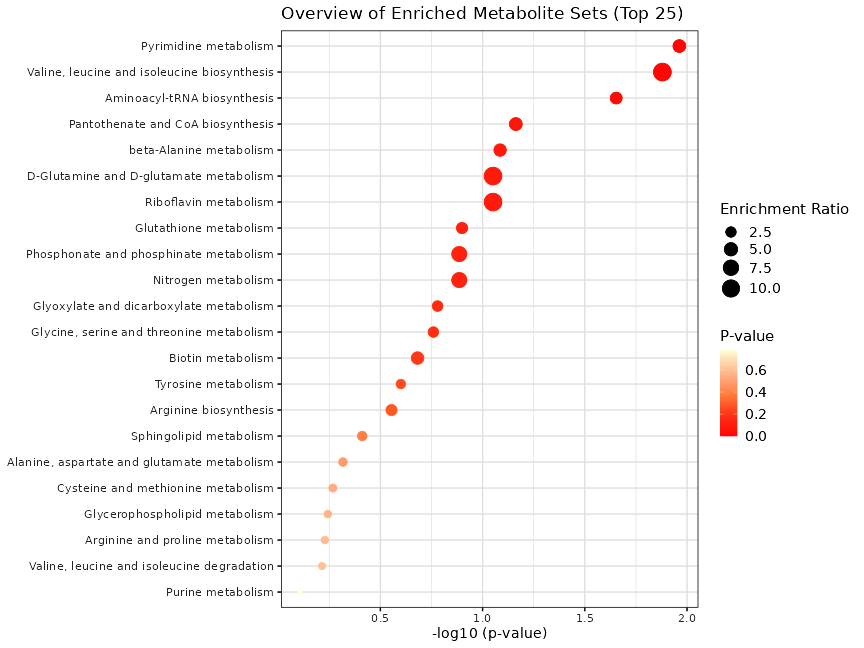


**Supplementary Figure 6 Enrichment of differential metabolites in female and male silkworms**

(A) Enrichment of common differential metabolites in all silkworms;(B) Enrichment of specific differential metabolites in females;(C) Enrichment of specific differential metabolites in males.
