# Supplementary material for: Metabolomics analysis of dietary restriction results in a longer lifespan due to alters of amino acid levels in larval hemolymph of Bombyx mori
Source: Sci Rep. 2023 Apr 26;13:6828. doi: 10.1038/s41598-023-34132-9 (PMC10133320; doi:10.1038/s41598-023-34132-9)
Supplement: Supplementary file 3 — Supplementary Tables. [file 41598_2023_34132_MOESM3_ESM.docx]

**Metabolomics Analysis of Dietary Restriction Results in a Longer Lifespan Due to Alters of Amino Acid Levels in Larval Hemolymph of *Bombyx mori***

Meixian WANG ^1,2†^, Yichen SHEN ^3†^, Zhicheng TAN ^1†^, Ayinuer YASEN ^1,2^, Bingyan FAN^1,2^, Xingjia SHEN ^1,2^*

1. Jiangsu Key Laboratory of Sericultural Biology and Biotechnology, College of Biotechnology, Jiangsu University of Science and Technology, Zhenjiang 212100, Jiangsu, China
2. Key Laboratory of Silkworm and Mulberry Genetic Improvement, Ministry of Agriculture and Rural Affairs, Sericultural Research Institute, Chinese Academy of

Agricultural Sciences, Zhenjiang 212100, Jiangsu, China

3. Department of Plastic Surgery, the First Affiliated Hospital, School of Medicine，Zhejiang University, Hangzhou 310009, Zhejiang, China

^†^These authors contributed equally to this work

*corresponding author: shenxjsri@163.com

**Corresponding author:**

SHEN Xingjia (Ph.D.)

Jiangsu University of Science and Technology

College of Biotechnology

Key Laboratory of Silkworm and Mulberry Genetic Improvement

212100 Zhenjiang

Email:shenxjsri@163.com

**Supplementary Table 1 Taxonomy of differential metabolites**

| **Metabolites** | **Hits** | | **Members** | |
| --- | --- | --- | --- | --- |
| Organic acids | | 15 | | Betaine, citric acid, L-phenylalanine,L-asparagine, L-isoleucine, L-histidine, oxoadipic acid, N-acetyl-L-phenylalanine, L-glutamine, L-leucine, citrulline, ascorbic acid, asymmetric dimethylarginine, L-methionine sulfoxide, L-targinine |
| Organoheterocyclic compounds | | 9 | | Biotin, Ascorbic acid,riboflavin, pyroglutamic acid, urocanic acid, 5-hydroxyindoleacetic acid, imidazoleacetic acid, indole-3-propionic acid, piperidine |
| Benzenoids | | 8 | | Tyramine, 2-pyrocatechuic acid, 3-hydroxyanthranilic acid, salicylic acid, vanillin, taxifolin, epsilon-caprolactam, cyanin |
| Polyketides | | 5 | | Caffeic acid, 3,3′,4′, 5-tetrahydroxystilbene, aesculetin, cyanin, luteolin 7-glucoside |
| Fatty acyls | | 4 | | 3-hydroxymethylglutaric acid, succinic acid semialdehyde, oxoadipic acid |
| Nucleic acids | | 4 | | Thymine, uridine, uracil, ribothymidine |
| Organic nitrogen compounds | | 4 | | Histamine, spermine, spermidine, phosphorylcholine |
| Organic oxygen compounds | | 3 | | L-kynurenine, chlorogenic acid, gentisate aldehyde |
| Carbohydrates | | 2 | | Sorbitol, orotidine |
| Prenol lipids | | 1 | | Gamma-terpinene |

**Supplementary Table 2 41 unique differential metabolites in the male group**

| Query | Match | HMDB | PubChem | KEGG |
| --- | --- | --- | --- | --- |
| L-Glutamine | L-Glutamine | HMDB0000641 | 5961 | C00064 |
| 5-Oxo-L-Proline | Pyroglutamic acid | HMDB0000267 | 7405 | C01879 |
| Phosphocholine | Phosphorylcholine | HMDB0001565 | 8691 | C00588 |
| Uridine | Uridine | HMDB0000296 | 6029 | C00299 |
| Uracil | Uracil | HMDB0000300 | 1174 | C00106 |
| MDMA | NA | NA | NA | NA |
| Tyramine | Tyramine | HMDB0000306 | 5610 | C00483 |
| N-epsilon-Acetyl-L-lysine | N6-Acetyl-L-lysine | HMDB0000206 | 92832 | C02727 |
| Kaempferol 3-O-beta-D-Glucoside | Quercetin 3-O-rhamnoside 7-O-glucoside | NA | 135626263 | C19796 |
| Piceatannol | 3,3',4'5-Tetrahydroxystilbene | HMDB0004215 | 667639 | C05901 |
| N-Formylanthranilic acid | NA | NA | NA | NA |
| Propionylcarnitine | Propionylcarnitine | HMDB0000824 | 107738 | C03017 |
| Taxifolin | Taxifolin | METPA0191 | NA | C01617 |
| Caffeic acid | Caffeic acid | HMDB0001964 | 1549111 | C01481 |
| 4-Hydroxybenzaldehyde | 4-Hydroxybenzaldehyde | HMDB0011718 | 126 | C00633 |
| Cyanin | Cyanin | METPA1737 | NA | C08639 |
| L-Ascorbic acid | Ascorbic acid | HMDB0000044 | 54670067 | C01041 |
| Spermine | Spermine | HMDB0001256 | 1103 | C00750 |
| Cytidine | Cytidine | HMDB0000089 | 6253 | C00475 |
| O-Lauroyl-L-Carnitine | Dodecanoylcarnitine | HMDB0002250 | 168381 | NA |
| Chlorogenic acid | Chlorogenic acid | HMDB0003164 | 1794427 | C00852 |
| gamma-Terpinene | Gamma-terpinene | HMDB0005806 | 7461 | C09900 |
| L-Threonine | L-Threonine | HMDB0000167 | 6288 | C00188 |
| 9H-Xanthine | NA | NA | NA | NA |
| L-Valine | L-Valine | HMDB0000883 | 6287 | C00183 |
| N-Acetyltryptophan | N-acetyltryptophan | HMDB0013713 | 700653 | NA |
| L-Serine | L-Serine | HMDB0000187 | 5951 | C00065 |
| Quercetin 3-O-beta-D-Glucopyranoside | Quercetin 3-O-glucuronide | HMDB0029212 | 12004528 | NA |
| Luteolin 7-O-beta-D-Glucoside | Luteolin 7-glucoside | HMDB0035588 | 5280637 | C03951 |
| 2,3-Dihydroxybenzoic acid | 2-Pyrocatechuic acid | HMDB0000397 | 19 | C00196 |
| 3,5-Dichlorosalicylic acid | NA | NA | NA | NA |
| Dehydroascorbic acid | Dehydroascorbic acid | HMDB0001264 | 7786 | C05422 |
| Malic acid | Malic acid | HMDB0000744 | 525 | C03668 |
| 4-Hydroxy-L-Proline | 4-Hydroxy-L-proline | HMDB0006055 | 69248 | C01015 |
| Isoscopoletin | NA | NA | NA | NA |
| Phosphatidylinositol lyso 20:4 | NA | NA | NA | NA |
| 2-Hydroxycinnamic acid | 2-Hydroxycinnamic acid | HMDB0002641 | 637540 | C01772 |
| Gentisic acid | Gentisic acid | HMDB0000152 | 3469 | C00628 |
| D-Biotin | Biotin | HMDB0000030 | 171548 | C00120 |
| 7-Methylguanine | 7-Methylguanine | HMDB0000897 | 11361 | C02242 |
| Riboflavin | Riboflavin | HMDB0000244 | 493570 | C00255 |

**Supplementary Table 3 28 unique differential metabolites in female**

| Query | Match | HMDB | PubChem | KEGG |
| --- | --- | --- | --- | --- |
| beta-Alanine | Beta-Alanine | HMDB0000056 | 239 | C00099 |
| Prantschimgin | NA | NA | NA | NA |
| Glycine Betaine | Betaine | HMDB0000043 | 247 | C00719 |
| L-Isoleucine | L-Isoleucine | HMDB0000172 | 6306 | C00407 |
| Benzamide | Benzamide | HMDB0004461 | 2331 | C09815 |
| Spermidine | Spermidine | HMDB0001257 | 1102 | C00315 |
| 6-Aminohexanoic acid | Aminocaproic acid | HMDB0001901 | 564 | C02378 |
| N,N-Dimethylglycine | Dimethylglycine | HMDB0000092 | 673 | C01026 |
| Choline | Choline | HMDB0000097 | 305 | C00114 |
| 2,5-Dihydroxybenzaldehyde | Gentisate aldehyde | HMDB0004062 | 70949 | C05585 |
| Tris(1-chloro-2-propyl)phosphate | NA | NA | NA | NA |
| L-Kynurenine | L-Kynurenine | HMDB0000684 | 161166 | C00328 |
| DL-Methionine sulfoxide | Methionine sulfoxide | HMDB0002005 | 847 | C02989 |
| 3-Hydroxy-DL-kynurenine | Hydroxykynurenine | HMDB0000732 | 89 | C02794 |
| 2-Pyrrolidinone | 2-Pyrrolidinone | HMDB0002039 | 12025 | C11118 |
| Acetoin | Acetoin | HMDB0003243 | 4068 | C00466 |
| N-Benzoylglycine | Hippuric acid | HMDB0000714 | 464 | C01586 |
| Pyridoxine | Pyridoxine | HMDB0000239 | 1054 | C00314 |
| 4-formyl Indole | NA | NA | NA | NA |
| D-Glucitol | Sorbitol | HMDB0000247 | 5780 | C00794 |
| Acetylcholine Chloride | Acetylcholine | HMDB0000895 | 6060 | C01996 |
| Citric acid | Citric acid | HMDB0000094 | 311 | C00158 |
| 5-Hydroxyindoleacetic acid | 5-Hydroxyindoleacetic acid | HMDB0000763 | 1826 | C05635 |
| vanillin | Vanillin | HMDB0012308 | 1183 | C00755 |
| D-Ribitol | NA | NA | NA | NA |
| PS(18:1(9Z)/0:0) | NA | NA | NA | NA |
| L-NMMA | L-Targinine | HMDB0029416 | 132862 | C03884 |
| 3-Indolepropionic acid | Indole-3-propionic acid | HMDB0002302 | 3744 | NA |

**Supplementary Table 4 33 differential metabolites appeared simultaneously in male and female groups**

| Query | Match | HMDB | PubChem | KEGG |
| --- | --- | --- | --- | --- |
| L-Cystathionine | L-Cystathionine | HMDB0000099 | 439258 | C02291 |
| Cyclohexylammonium | NA | NA | NA | NA |
| L-Lysine | L-Lysine | HMDB0000182 | 5962 | C00047 |
| Putrescine | Putrescine | HMDB0001414 | 1045 | C00134 |
| Thymine | Thymine | HMDB0000262 | 1135 | C00178 |
| Ribothymidine | Ribothymidine | HMDB0000884 | 445408 | NA |
| L-Pipecolic acid | L-Pipecolic acid | HMDB0000716 | 439227 | C00408 |
| Histamine | Histamine | HMDB0000870 | 774 | C00388 |
| N-Omega-Acetylhistamine | N-Acetylhistamine | HMDB0013253 | 69602 | C05135 |
| 2-Methylguanosine | 2-Methylguanosine | HMDB0005862 | 6474236 | NA |
| Guanosine | Guanosine | HMDB0000133 | 6802 | C00387 |
| Citrulline | Citrulline | HMDB0000904 | 9750 | C00327 |
| L-Phenylalanine | L-Phenylalanine | HMDB0000159 | 6140 | C00079 |
| Piperidine | Piperidine | HMDB0034301 | 8082 | C01746 |
| Imidazoleacetic acid | Imidazoleacetic acid | HMDB0002024 | 96215 | C02835 |
| epsilon-Caprolactam | epsilon-Caprolactam | METPA0843 | NA | C06593 |
| L-Histidine | L-Histidine | HMDB0000177 | 6274 | C00135 |
| L-Tyrosine | L-Tyrosine | HMDB0000158 | 6057 | C00082 |
| Phenacylamine | 2-Aminoacetophenone | HMDB0032628 | 11952 | NA |
| Hypoxanthine | Hypoxanthine | HMDB0000157 | 790 | C00262 |
| 3-Hydroxyanthranilic acid | 3-Hydroxyanthranilic acid | HMDB0001476 | 86 | C00632 |
| Inosine | Inosine | HMDB0000195 | 6021 | C00294 |
| L-Arginine | L-Arginine | HMDB0000517 | 6322 | C00062 |
| Orotidine | Orotidine | HMDB0000788 | 92751 | C01103 |
| Galactitol | Galactitol | HMDB0000107 | 11850 | C01697 |
| 3-Hydroxy-3-Methylglutaric acid | 3-Hydroxymethylglutaric acid | HMDB0000355 | 1662 | C03761 |
| Succinic acid semialdehyde | Succinic acid semialdehyde | HMDB0001259 | 1112 | C00232 |
| 2-Oxoglutaric acid | Oxoglutaric acid | HMDB0000208 | 51 | C00026 |
| Esculetin | Aesculetin | HMDB0030819 | 5281416 | C09263 |
| Oxoadipic acid | Oxoadipic acid | HMDB0000225 | 71 | C00322 |
| N-Acetyl-L-Phenylalanine | N-Acetyl-L-phenylalanine | HMDB0000512 | 74839 | C03519 |
| 5-Aminosalicylic acid | Mesalazine | HMDB0014389 | 4075 | NA |
| Aminohippuric acid | 4-Aminohippuric acid | HMDB0001867 | 2148 | D06890 |
